# Conflicting clinical and radiological management decisions

**DOI:** 10.1002/ccr3.3698

**Published:** 2021-01-09

**Authors:** Tamer Mohamed Zaalouk, Zouheir Ibrahim Bitar, Ossama Sajeh Maadarani

**Affiliations:** ^1^ Critical Care Unit Ahmadi Hospital Kuwait Oil Company Fahaheel Kuwait

**Keywords:** chronic, encapsulated, intracranial, haematoma

## Abstract

Gliosis with hemorrhagic transformation is a late reported complication of stroke. Sometimes there is a big discrepancy between clinical and radiological diagnosis, and clinical decisions must be multi‐aspect decisions and not dependent on a single discrepant investigation result.

## Graphical abstract

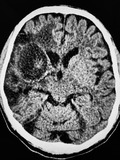

## 1 QUESTIONS AND TEXT

Q1 What is your diagnosis for these CT and MRI images?

Q2 What is your plan of management?

**FIGURE 1:**
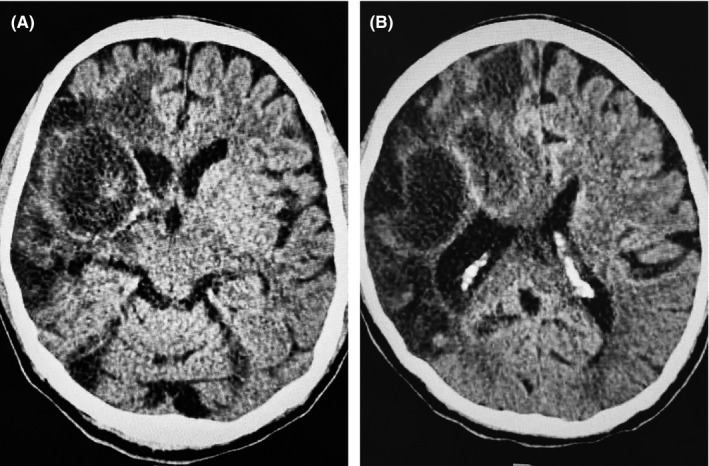
A, B CT with contrast images

**FIGURE 2:**
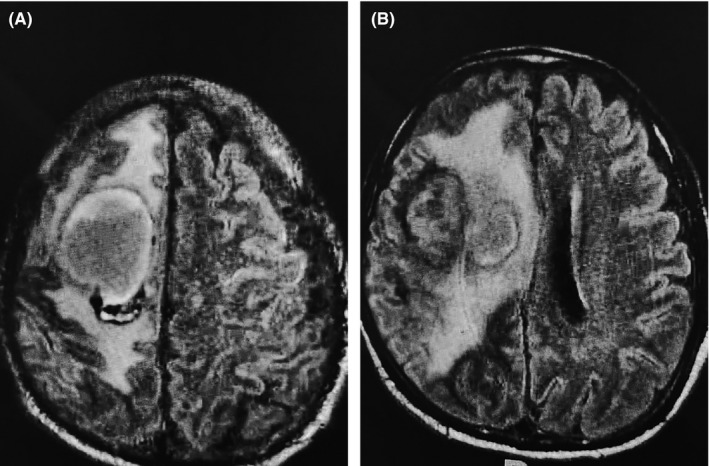
A, B MRI T2 Flair images

A 48‐year‐old Asian man with a history of ischemic cardiomyopathy with a low ejection fraction (EF 15%) received thrombolytic therapy two months before presentation to our hospital due to a right middle cerebral artery stroke.^1^, ^2^ He presented to our critical care unit with a low cardiac output state requiring inotropic support. A follow‐up CT brain in our unit (Figure 1A,B) showed multiple hypodense areas with hemorrhagic transformation and mass effect. An MRI confirmed chronic encapsulated intracerebral hematoma (CEIH) with surrounding edema (Figure 2A,B). The neurosurgeon's decision is based on only reviewing the CT and MRI images, to start mannitol trying to decrease the brain edema and to refer patient to the neurosurgery center for decompressive craniotomy.

Surprisingly, the patient Glasgow coma scale (GCS) was 15 with left hemiplegia. Even with no mannitol, the patient remained stable with the same GCS of 15.

## CONFLICT OF INTEREST

Not declared.

## AUTHOR CONTRIBUTIONS

TZ and OM: collected the information and drafted the manuscript. ZB: revised and approved the final manuscript. Our working website is www.kockw.com, Kuwait Oil Company, Ahamdi hospital.

## CONSENT

Informed consent was obtained from the patient for the publication of this clinical image.
